# GFP-Tagged E^rns^ in Bungowannah Pestivirus: A Tool for Viral Tracking and Functional Studies

**DOI:** 10.3390/v18020263

**Published:** 2026-02-20

**Authors:** Sara Ezzat, Matthias Schweizer

**Affiliations:** 1 Institute of Virology and Immunology, Länggass-Str. 122, POB, CH-3001 Bern, Switzerland; sara.ezzat@unibe.ch; 2 Department of Infectious Diseases and Pathobiology, Vetsuisse Faculty, University of Bern, CH-3012 Bern, Switzerland; 3 Graduate School for Cellular and Biomedical Sciences, University of Bern, CH-3012 Bern, Switzerland

**Keywords:** pestivirus, bovine viral diarrhea virus (BVDV), Bungowannah pestivirus (BuPV), fusion protein, green fluorescent protein (GFP), envelope glycoprotein, secreted viral RNase

## Abstract

Pestiviruses, such as bovine viral diarrhea virus (BVDV) or classical swine fever virus (CSFV), are members of the family *Flaviviridae* and infect a broad range of species, causing significant economic losses in livestock. A unique feature of pestiviruses is the E^rns^ protein, which is part of the glycoprotein complex at the surface of the virion, but it is also secreted as an RNase that functions as an interferon (IFN) antagonist. This dual nature makes E^rns^ a particularly complex and multifunctional protein, highlighting its importance for understanding pestivirus biology. Bungowannah pestivirus (BuPV) was reported to exhibit high genetic plasticity, making it suitable for engineering recombinant tools. In this study, we generated a recombinant BuPV expressing green fluorescent protein (GFP) fused to the N-terminus of the E^rns^ protein from BVDV. The GFP-E^rns^ fusion was detected by fluorescence microscopy and remained stable across five serial passages. The recombinant virus infected all tested mammalian cell lines but replicated more slowly than the parental BuPV stock. RNase activity assays confirmed retention of enzymatic function. These results demonstrate stable expression, broad infectivity, and preserved activity of GFP-E^rns^ in the recombinant BuPV, indicating that this might be a useful tool for further investigations on pestivirus pathogenesis.

## 1. Introduction

Belonging to the family *Flaviviridae*, pestiviruses are enveloped, positive-sense, single-stranded RNA viruses. This genus includes several economically important pathogens, such as bovine viral diarrhea virus (BVDV-1 and -2; species *Pestivirus bovis* and *Pestivirus tauri*), classical swine fever virus (CSFV; species *Pestivirus suis*), and Border disease virus (BDV; species *Pestivirus ovis*) [1]. Their impact on livestock health and productivity has made them the focus of extensive research [2,3,4], particularly regarding their replication mechanisms and interactions with the host immune system [5,6,7,8]. The genome of pestiviruses is composed of a single open reading frame (ORF) flanked by two untranslated regions (UTR) at the 5′- and 3′ ends. The ORF is translated into one polyprotein, which will be cleaved by viral and cellular proteases to generate at least 12 different viral proteins. Among those are two interferon (IFN) antagonists, i.e., N^pro^, a nonstructural protein, and the envelope glycoprotein E^rns^, which are both considered hallmarks of pestiviruses. N^pro^ promotes immune evasion by targeting IFN regulatory factor 3 (IRF3) for proteasomal degradation, thereby suppressing IFN production in infected cells [9,10]. By contrast, E^rns^ functions as a secreted endoribonuclease that degrades viral RNA to prevent its recognition by Toll-like receptors (TLR) 3 and 7, key sensors of viral double- (ds) and single-stranded (ss) RNA, respectively [11,12,13]. In addition to its role in immune modulation, E^rns^ is also an essential structural glycoprotein of the virion, along with E1 and E2 [12]. While E1 and E2 mediate host cell entry through receptor binding and membrane fusion, E^rns^ is implicated in the initial attachment of the virus to the host cell surface via interactions with heparan sulfate [14,15,16]. This dual role of E^rns^—as both an immune evasion factor and a structural component—suggests a complex intracellular trafficking and processing pathway that remains incompletely understood. One proposed model suggests that following endocytosis of E^rns^, it may act locally to degrade immunostimulatory RNA before TLR-mediated detection occurs, e.g., in case free viral RNA is released within endolysosomes [13,17]. However, the precise mechanisms governing E^rns’^ trafficking and secretion, as well as its spatial regulation during infection, are still unclear.

To facilitate the study of the dynamics of E^rns^ expression and function, a genetically tagged virus expressing a fluorescent fusion protein such as GFP-E^rns^ would be an invaluable tool. We used Bungowannah pestivirus (BuPV; species *Pestivirus australiaense*), known for its genomic plasticity and broad cell tropism [18,19,20], as an ideal platform to engineer a pestivirus expressing a recombinant GFP-tagged E^rns^ protein. Previous work has demonstrated that GFP can be fused to the N-terminus of the soluble E^rns^ protein expressed as a single protein, disturbing neither its function as an IFN antagonist nor the GFP signal [21]. This positioning avoids interference with C-terminal domains involved in proteolytic processing and heparan sulfate binding, both of which are critical for viral replication and entry [13,22]. In this study, we report on the stable generation and characterization of BuPV particles labeled with a fluorophore fused to E^rns^ without interfering with its role as surface glycoprotein and RNase, enabling visualization of infection dynamics, providing a powerful tool for investigating E^rns^ trafficking, secretion, and interactions with host cells.

## 2. Materials and Methods

### 2.1. Cells and Parental Virus

Swine kidney cell line SK-6, Vero, and HEK-293a were previously used at our institute [9,23,24], and they were grown in Dulbecco’s modified Eagle medium (DMEM) supplemented with 10% BVDV-free fetal calf serum (FCS) (PAA Laboratories; Lucerna-Chem AG, Lucerne, Switzerland), 1% non-essential amino acids (Gibco; Thermo Fisher Scientific, Reinach, Switzerland), and 100 units/mL of penicillin and 100 μg/mL of streptomycin at 37 °C and 5% CO_2_. The same conditions were applied for MDBK ([25]; American Type Culture Collection ATCC, Manassas VA, USA) and KOP-R except that it was Minimal Essential Medium (MEM) supplemented with 10% FCS, 1% non-essential amino acids, and penicillin/streptomycin. The KOP-R cells [19] were kindly provided by Martin Beer (FLI Riems, Germany). The parental (wild type) BuPV was kindly provided by M. Beer (FLI Riems, Germany, with the permission of P.D. Kirkland, Elizabeth Macarthur Agricultural Institute, Menangle, New South Wales, Australia) [18].

### 2.2. Bacterial Cloning of pCI GFP-BVDV ncp7 E^rns^

To flank the cassette “GFP-BVDV ncp7 E^rns^”, which represents Fragment 2, with 100 bp overlapping for the yeast cloning, the plasmid GFP-ncp7 E^rns^ designed by Carmela Lussi [21] was amplified by PCR with the following primers: GFP-ncp7_E^rns^_Forward (5′-GTACCGGTAGGGTCCATGGTGAGCAAGGGCGAG-3′) and GFP-ncp7_E^rns^_Reverse (5′-TGGGCAGTAAGGACTAGCATATGCCCCAAACCATG-3′). The vector pCI was linearized by PCR with the following primers: Open_pCI_GFP-ncp7_E^rns^_Reverse (5′-GCCCTTGCTCACCATGGACCCTACCGGTACCAC-3′) and Open_pCI_GFP-ncp7_E^rns^_Forward (5′-TTTGGGGCATATGCTAGTCCTTACTGCCCAGTGG-3′) and DpnI-digested. Both digested products were run on a 1% agarose gel, and the bands were cut and purified using the Nucleospin Gel and PCR clean-up kit following the manufacturer’s recommendations (Macherey-Nagel, Oensingen, Switzerland). Lastly, the insert was cloned in the linearized vector using the “In-Fusion HD cloning kit” from Clontech (Takara Bio Company, Saint-Germain-en-Laye, France) by following the manufacturer’s instructions. Briefly, the PCR-amplified fragment and linearized vector were ligated at a 1:2 molar ratio for 15 min at 50 °C, followed by bacterial transformation. Plasmid sequences were confirmed by Sanger sequencing. Primers and sequencing were produced and performed by Microsynth (Balgach, Switzerland).

### 2.3. Generation of cDNA Fragments and In-Yeast Homologous Recombination for Virus Production

Full-length viral cDNAs for infectious clone assembly were generated by transformation-associated recombination (TAR) cloning following the methods described by Thao et al. [26]. Overlapping DNA fragments covering the entire viral genome were designed using the primer pairs listed in Table 1. Each fragment was flanked with at least 50 bp of homology to its corresponding upstream and downstream fragments and synthesized via one-step RT-PCR using the SuperScript™ IV system (Invitrogen; Thermo Fisher Scientific, Waltham, MA, USA). PCR products were visualized by agarose gel electrophoresis, purified using the Nucleospin Gel and PCR clean-up kit following manufacturer’s recommendations (Macherey-Nagel), and quantified at 280 nm with a NanoDrop spectrophotometer (Thermo Fisher Scientific; Witec AG, Sursee, Switzerland) to ensure equimolar pooling. In parallel, the TAR cloning vector pCC1BAC-His3 was similarly amplified and purified. All cDNA fragments and the linearized TAR vector were co-transformed into *Saccharomyces cerevisiae* strain VL6-48N via a lithium acetate/PEG transformation protocol. Transformed yeast colonies were selected on SD-His agar plates, and assemblies were screened by PCR over the junctions to verify the correct integration of all fragments. Colonies containing correctly assembled constructs were cultured for plasmid minipreps, amplified by EquiPhi29 (Thermo Fisher Scientific), digested by PacI (New England Biolabs, Ipswich, MA, USA; BioConcept Ltd., Allschwil, Switzerland), and in vitro transcribed by the T7 RiboMax™ Large Scale RNA Production System (Promega AG, Dübendorf, Switzerland).

### 2.4. Virus Rescue and Propagation

To rescue the virus, SK-6 cells (3 × 10^5^ cells per well) were seeded into 6-well plates one day prior to transfection. Transfection was carried out using the TransIT^®^-mRNA Transfection Kit (Mirus Bio; LabForce AG, Muttenz, Switzerland) following the manufacturer’s instructions. Briefly, 1.5 μg of in vitro-transcribed RNA was diluted in a final volume of 30 μL of nuclease-free water, and 250 μL of Opti-MEM™ Reduced Serum Medium (Gibco) was added. To this solution, 5 μL of mRNA Boost Reagent and 5 μL of TransIT-mRNA Reagent were sequentially added. The mixture was gently mixed and incubated at room temperature for 2–5 min. The transfection complex was then added dropwise to the cells, and the plate was gently rocked to distribute the reagents evenly. Cells were incubated at 37 °C in a humidified atmosphere with 5% CO_2_ for 72 h in the presence of the transfection complexes.

For virus propagation, 4 × 10^6^ SK-6 cells were seeded in a T-75 flask and infected with the rescued virus at a multiplicity of infection (MOI) of 0.01 in 10 mL of serum-free DMEM. After 1 h of incubation at 37 °C, the inoculum was removed, cells were washed with 10 mL of PBS, and 12 mL of fresh DMEM supplemented with 10% FCS was added. The culture was incubated for 5 days and the supernatant was cleared by centrifugation at 400× *g* for 10 min. Viral titers were determined on SK-6 cells by immunoperoxidase staining [27] using the monoclonal pan-pestivirus antibody RAE2020 (APHA Scientific, Weybridge, UK) as primary antibody, and titers were determined using the method of Reed and Muench [28]. Results were expressed as 50% tissue culture infectious doses (TCID_50_) per ml.

### 2.5. Virus Growth Kinetics and RT-qPCR

SK-6 cells were seeded two hours prior to viral infection in a 24-well plate at a density of 2.4 × 10^6^ cells per plate. Cells were washed once with PBS and inoculated with viruses (MOI of 0.1) in DMEM without FCS. After one hour, the virus-containing supernatant was removed, and cells were washed three times with PBS and DMEM 10% FCS. Cell culture supernatants were collected at the indicated time points after infection. For the RT-qPCR, viral RNA from the supernatant was extracted using the QIAamp Viral RNA kit (Qiagen AG, Hombrechtikon, Switzerland). After purification, a one-step reverse transcription polymerase chain reaction (RT-qPCR) was done by the TaqMan™ Fast Virus 1-Step Master Mix (Applied Biosystems™; Thermo Fisher Scientific) with the following primers and probe: Bung-395-frw_N^pro^ (5′-TTAATACATATGGAGGGAGTGAGGAA-3′) and Bung-396-rev_N^pro^ (5′-TCGCCAAAAATTGGTCTCATT-3′) and FAM-labeled probe Bung-397-probe_N^pro^ (5′-ACCCACGCCGCCACCAGG-3′) targeting the N^pro^ region. Amplification parameters were used following the manufacturer’s instructions. Briefly, the RNA was reverse transcribed at 50 °C for 5 min before activation of the polymerase at 95 °C for 20 s, followed by 45 cycles of denaturation at 95 °C for 3 s and annealing and extension at 60 °C for 30 s.

### 2.6. Mass Spectrometry Sample Preparation and Analysis

SDS-PAGE separation was performed on cell lysates infected by BuPV-GFP ncp7 E^rns^. Two distinct protein bands corresponding to the approximate molecular weights of interest were excised manually from a PVDF membrane. Then, the “Core Facility Proteomics & Mass Spectrometry” of the University of Bern processed all the following steps: the PVDF membrane pieces were digested for 6 h at 37 °C with 15 μL of 0.1 μg/μL sequencing grade trypsin (Promega) in 50 mM Tris-HCl pH 8/10% acetonitrile after performing membrane blocking with 0.5% (*w*/*v*) polyvinylpyrolidone (Sigma, Buchs, Switzerland) in 0.1% (*v*/*v*) acetic acid for 30 min at 40 °C, followed by washing five times in neat water, reduction and alkylation of proteins in 50 mM dithioerythritol/50 mM iodoacetamide for 30 min at 37 °C each, and final washing three times with 50 mM Tris-HCl pH 8. The digested supernatant was combined with 10 μL of 20% formic acid extraction solution for 15 min at room temperature. An aliquot of 2 μL was analyzed on an LC-MS/MS system consisting of a nanoElute2 UPLC and a timsTOF HT mass spectrometer (Bruker, Bremen, Germany) using the same column setup and acquisition method as described [29].

The mass spectrometry facility analyzed the data with Fragpipe v22 [30] against a concatenated protein sequence database of the Bungowannah pestivirus, UniprotKB *Sus scrofa* (version 2024_9), and common contaminant sequences (keratins, trypsin, etc.). Search parameters included precursor and fragment tolerances of ±20 ppm/±0.05 Da, up to two isotope errors, three allowed missed cleavages, fixed carbamidomethylation of cysteines, and variable oxidation of methionines and protein N-terminal acetylation, respectively. Identifications were evaluated with Percolator using msbooster spectrum prediction of Prosit_2019_iRT and Prosit_2023_intensity_timsTOF for retention time and spectrum modeling at a false discovery rate of 1%. Furthermore, all proteins not identified with at least 2 unique peptide sequences were rejected.

### 2.7. Western Blot

Western blot was performed on a nitrocellulose membrane as described [31], except that the samples were heated at 70 °C for 5 min and loaded on precast polyacrylamide SurePAGE™ Bis-Tris 10% gels (GenScript, Piscataway, NJ, USA; Witec AG, Sursee, Switzerland). The gels were run for 20 min at 80 V and 50 min at 140 V in MOPS buffer. As chemiluminescence substrate, WesternBright ECL and Quantum HRP substrate (Advansta Inc., San Jose, CA, USA; Witec AG) were used. For staining of GFP and E^rns^ proteins, a primary mouse monoclonal antibody against GFP (B-2 clone, Santa Cruz Animal Health, kindly provided by Thomas Kaufmann, Institute of Pharmacology, University of Bern, Bern, Switzerland) or E^rns^ (RAE0823, APHA Scientific) was used. As a housekeeping protein, β-actin was detected with a mouse monoclonal anti-β-actin antibody (Sigma). As a secondary antibody, peroxidase-conjugated AffiniPure Donkey anti-mouse IgG (Jackson ImmunoResearch Europe Ltd., Ely, UK; Milan Analytica AG, Rheinfelden, Switzerland) was used to reveal both primary antibodies.

### 2.8. Fluorescence Microscopy

For fluorescence analyses, 1 × 10^5^ SK-6 cells per well were grown on glass coverslips with thickness No. 1.5H (Paul Marienfeld GmbH & Co. KG, Lauda-Königshofen, Germany) in a 24-well plate. After adherence, cells were infected with an MOI of 3 in DMEM without FCS for one hour. After incubation, SK-6 cells were washed twice with PBS before cultivation in DMEM 10% FCS for 24 h. The next day, cells were fixed with 4% formalin for 15 min. Nuclei were stained with DAPI present in the mounting medium (Invitrogen; Thermo Fisher Scientific). Visualization was performed using a Nikon Ti2 Cicero Spinning Disc (Nikon, Egg, Switzerland) with a 20× air objective. Image processing was done with Fiji (ImageJ v1.54j) [32].

### 2.9. Ultrafiltration, Quantification of Soluble E^rns^ and RNase Activity Assay

To remove viral particles, cell culture supernatants were filtrated using Pierce™ Protein Concentrators PES, 100 K MWCO, 0.5–100 mL (Thermo Scientific^TM^, Thermo Fisher Scientific) according to the manufacturer’s protocol. The flowthrough of the 100 K MWCO was collected, and secreted E^rns^ was concentrated by using a Pierce™ Protein Concentrators PES, 50 K MWCO, 0.5–100 mL (Thermo Scientific^TM^), and washed twice with Tris-acetate buffer (pH 5.5). Residual viral contamination was assessed by titration. The retentate from the second ultrafiltration contained the concentrated soluble E^rns^, which was quantified using a BVDV Ag/Serum Plus ELISA kit (IDEXX; IDEXX Switzerland GmbH, Bern-Liebefeld, Switzerland) using purified E^rns^ as a standard [21]. For the RNase activity assay, a 300 bp dsRNA substrate from the 5′-UTR of BVDV strain Ncp7 was generated by annealing in vitro-transcribed complementary ssRNA as described [21]. The dsRNA and ultrafiltrated E^rns^ were diluted in Tris-acetate buffer (pH 5.5). Equal volumes of enzyme and substrate were incubated at 37 °C for one hour in a 10 μL reaction volume. Products were mixed with loading dye and separated on a 1% agarose gel.

## 3. Results

### 3.1. Design of GFP-ncp7 E^rns^ Bungowannah Pestivirus

**Figure 1:**
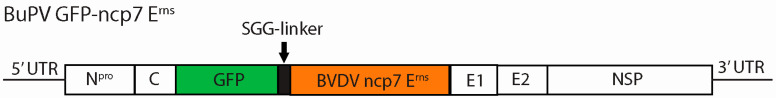
Schematic representation of the recombinant BuPV expressing GFP-tagged BVDV ncp7 E^rns^. The construct design involves the insertion of green fluorescent protein (GFP) at the N-terminus of the E^rns^ protein derived from BVDV ncp7. A short flexible linker composed of serine (S) and glycine (G) residues was introduced between GFP and E^rns^ to reduce possible interference with protein function. In the schematic, BuPV nonstructural and structural proteins are depicted in white, GFP in green, the linker in black, and the BVDV ncp7 E^rns^ in orange.

To enable real-time visualization of E^rns^ expression, localization, and trafficking dynamics, we generated a recombinant BuPV genome encoding a GFP-tagged version of E^rns^. Specifically, the design involved fusing GFP to the N-terminus of E^rns^ derived from the BVDV strain ncp7. It has been demonstrated that this N-terminal fusion does not interfere with the RNase activity of E^rns^, indicating that the structural and functional integrity of the protein is preserved despite the addition of the fluorescent tag [21]. Importantly, BuPV has been shown to exhibit considerable genetic plasticity, particularly in tolerating modifications and substitutions within its structural protein coding regions [19,20]. Leveraging this flexibility, we utilized the BuPV genome as a modular scaffold to replace the native BuPV E^rns^ coding sequence with a synthetic cassette encoding GFP-BVDV ncp7 E^rns^. This strategy allowed us to combine the well-characterized enzymatic properties of BVDV E^rns^ with the genetic tractability of BuPV (Figure 1). In addition, expression of BVDV E^rns^ in the recombinant virus enabled detection with BVDV-specific antibodies and by ELISA, methods that are currently not available for the native BuPV E^rns^. Lastly, this construct provided a useful tool to track E^rns^ expression and assess its localization during infection.

### 3.2. Stability of GFP Expression and Replication Kinetics

**Figure 2:**
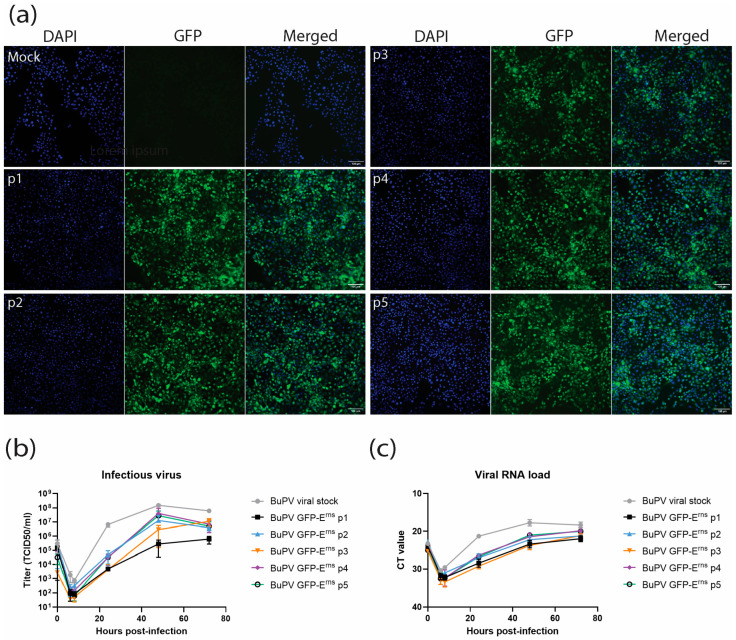
Stability and replication of recombinant BuPV GFP-BVDV ncp7 E^rns^ across serial passages. (**a**) SK-6 cells were infected at a multiplicity of infection (MOI) of 3 with the recombinant BuPV GFP-BVDV ncp7 E^rns^ and monitored at 2 days post-infection across five serial passages (p1–p5). Mock-infected cells served as a negative control. Cells were fixed and stained with DAPI (blue) to visualize nuclei, and GFP fluorescence (green) was used to assess expression of the tagged E^rns^ protein. Representative images show consistent GFP expression across passages. Scale bar: 100 μm. (**b**) Viral replication kinetics of the BuPV GFP-BVDV ncp7 E^rns^ construct (p1–p5) in SK-6 cells compared to the parental BuPV viral stock in SK-6 cells. Cells were infected at an MOI of 0.1 and supernatants were collected at 6, 8, 24, 48, and 72 h post-infection. Viral titers were determined by TCID_50_ assay. Data represents mean values from three technical replicates. (**c**) Quantification of viral RNA load in infected SK-6 cells at the same time points as in (**b**). Viral RNA was extracted and quantified by RT-qPCR, expressed as CT values. BuPV viral stock and BuPV GFP-BVDV ncp7 E^rns^ (p1–p5) were compared to assess RNA replication efficiency. Data represents mean values from three technical replicates.

Using transformation-associated recombination (TAR) cloning, cDNA fragments were assembled to construct a recombinant BuPV genome encoding a GFP-tagged BVDV E^rns^. The full-length viral RNA was then transcribed in vitro and transfected into SK-6 cells, leading to the rescue of infectious viruses. To assess the genetic stability of a GFP-expressing BuPV construct, we serially passaged the virus five times on SK-6 cells. The initial viral stock (p0) was derived from the supernatant of transfected cells, and subsequent passages (p1–p5) were generated from sequential infections using cell culture supernatants. Fluorescence microscopy demonstrated persistent GFP expression across all passages (Figure 2a), suggesting that the inserted reporter gene was stably maintained in the viral genome.

To evaluate the impact of the GFP insertion on replication fitness, we compared the replication kinetics of the GFP-expressing virus to those of the parental BuPV. Viral growth curves indicated that the GFP-tagged virus replicated more slowly (Figure 2b,c). Notably, at 6 h post-infection, the GFP-tagged virus exhibited a significantly lower titer compared to the viral stock (Supplementary Material Table S1).

### 3.3. Tropism of GFP-Tagged BuPV

**Figure 3:**
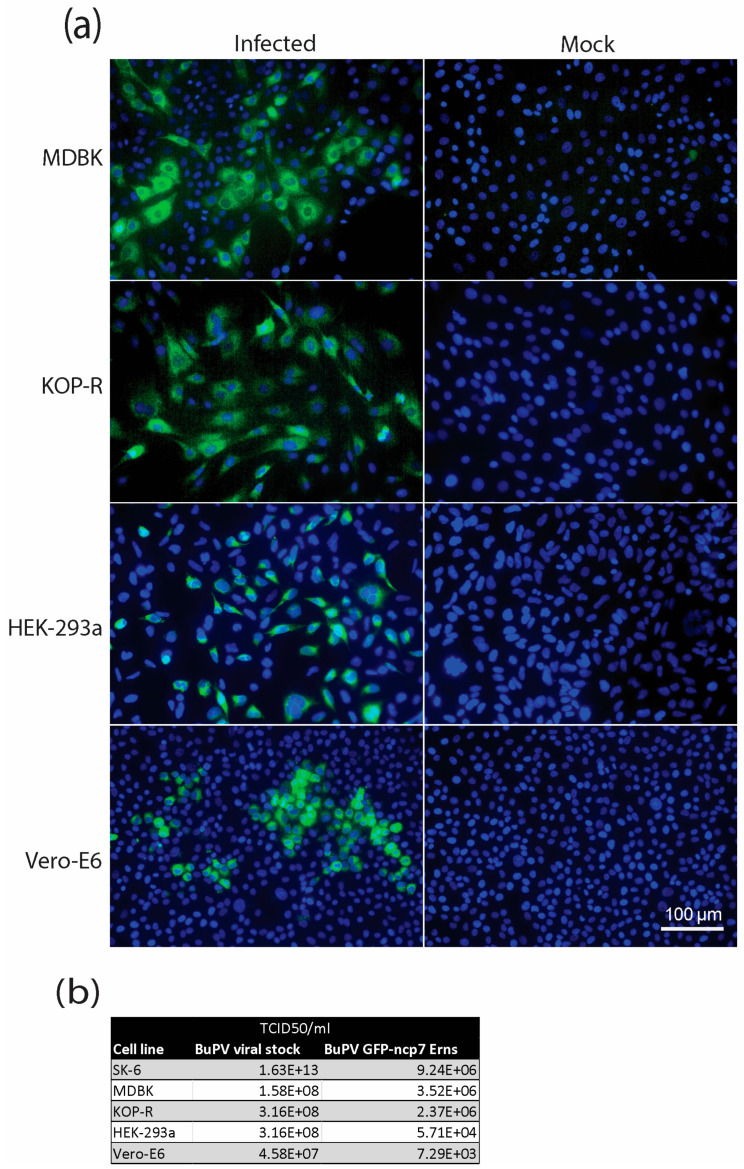
Cell tropism and replication efficiency of recombinant BuPV GFP-BVDV ncp7 E^rns^ in various mammalian cell lines. (**a**) MDBK, KOP-R, HEK-293a, and Vero-E6 cells were infected with BuPV GFP-BVDV ncp7 E^rns^ p1 at a multiplicity of infection (MOI) of 3 and fixed two days post-infection. GFP fluorescence (green) indicates viral protein expression, while nuclei were stained with DAPI (blue). Mock-infected cells served as negative controls for each cell line. Scale bar: 100 μm. (**b**) Viral titers from the supernatants of infected cells were determined by TCID_50_ assay on SK-6 cells. Data represents the mean titers from two independent experiments.

Given previous reports suggesting a broad host cell range for BuPV [18], we aimed to evaluate the cellular tropism of the recombinant GFP-tagged virus. To this end, a panel of cell lines representing different species—MDBK (bovine kidney cells), KOP-R (bovine esophagus cells), HEK-293a (human embryonic kidney cells), and Vero-E6 (African green monkey kidney cells)—were infected with the recombinant virus. GFP expression was monitored by fluorescence microscopy as a proxy for infection efficiency and transgene expression. This approach enabled a comparative assessment of BuPV infectivity across diverse cellular backgrounds and provided evidence that all tested cell lines were susceptible to the GFP reporter virus (Figure 3a).

To further evaluate viral replication, supernatants from the infected cell lines were collected and titrated on SK-6 cells using a TCID_50_ assay (Figure 3b). Detectable infectious titers in the supernatant confirmed productive viral replication and excluded the possibility of an abortive infection. The titration revealed that the viral stock exhibited higher titers than the recombinant BuPV expressing GFP-BVDV ncp7 E^rns^, indicating a reduction in replication efficiency in the engineered virus compared to the parental stock as observed in the parental SK-6 cell line (Figure 2). These results are consistent with the possibility that the insertion of the GFP-tagged E^rns^ may affect viral replication fitness. Notably, the rather large differences in TCID_50_ values observed between Figure 2b and Figure 3b might be attributable to variable experimental conditions, including the use of different MOIs (MOI of 0.1 and 3 in Figure 2b and Figure 3b, respectively), and fewer washing steps after infection with BuPV GFP-BVDV ncp7 E^rns^ of passage 1 in the latter experiment, i.e., a virus that was not yet fully adapted to SK-6 cells, in contrast to the parental BuPV.

### 3.4. GFP Is Attached to BVDV ncp7 E^rns^

**Figure 4:**
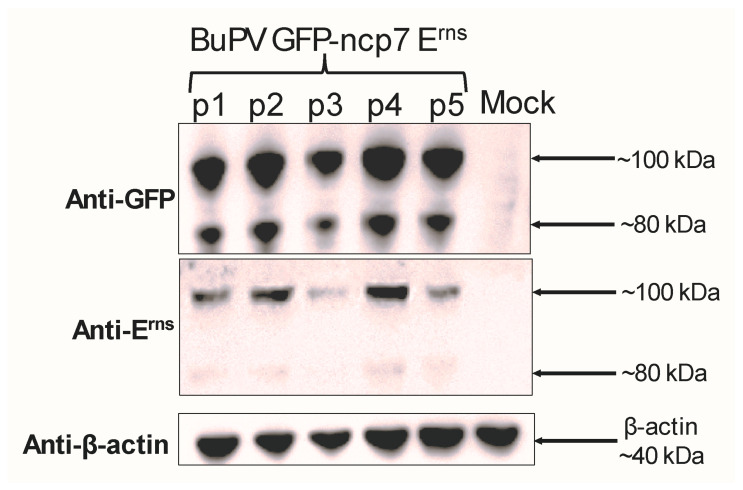
Western blot analysis of GFP-tagged BVDV ncp7 E^rns^ expression across virus passages. SK-6 cells were infected with BuPV GFP-BVDV ncp7 E^rns^ from passage 1 to passage 5 (p1–p5) at an MOI of 3. Cell lysates were collected 22 h post-infection and analyzed by SDS-PAGE followed by Western blotting. Membranes were probed with antibodies against GFP (top panel), E^rns^ (middle panel), and β-actin (bottom panel). A specific band corresponding to GFP-ncp7 E^rns^ (~80 kDa) was detected with both anti-GFP and anti-E^rns^ antibodies in all infected samples, confirming expression of the intact fusion protein. In addition, a second band with an apparent molecular weight of ~100 kDa was equally detected by both antibodies. No signal was observed in the mock-infected control. β-actin (~40 kDa) was used to verify equal protein loading. The uncropped Western blots are available in Supplementary Material Figure S1.

To confirm the successful attachment of GFP to the N-terminus of BVDV ncp7 E^rns^, SK-6 cells were infected with the recombinant BuPV GFP-BVDV ncp7 E^rns^ at an MOI of 3 and lysed 22 h post-infection for Western blot analysis. Using antibodies directed against GFP and E^rns^, two protein bands, one of the expected molecular weight (lower band) and another higher one (upper band) were detected with both antibodies, though the E^rns^ antibody only weakly detected the fusion proteins. This indicated that GFP and E^rns^ are expressed as a single fusion protein but show two different molecular weights (Figure 4), with the identity of the upper band being unknown.

To further investigate the nature of the two distinct bands observed by Western blot using anti-GFP and anti-E^rns^ antibodies (Figure 4), we excised the upper and lower bands directly from the PVDF membrane and submitted them for mass spectrometry analysis. This approach aimed to determine whether the bands corresponded to different forms of the GFP-ncp7 E^rns^ fusion protein and to explore potential post-translational modifications. Mass spectrometry confirmed the presence of GFP-ncp7 E^rns^ in both bands, with the lower band showing a higher number of spectral counts. The viral E1 protein was detected exclusively in the lower band, while the upper band contained only traces of GFP-ncp7 E^rns^ and no E1 (Table 2). These results suggest that the two bands represent distinct molecular forms of the construct. Although no direct evidence for glycosylation was found, the electrophoretic shift and disproportionate signal intensity in the upper band support the hypothesis that it may reflect a post-translationally modified variant.

### 3.5. GFP-ncp7 E^rns^ Function Is Still Maintained

**Figure 5:**
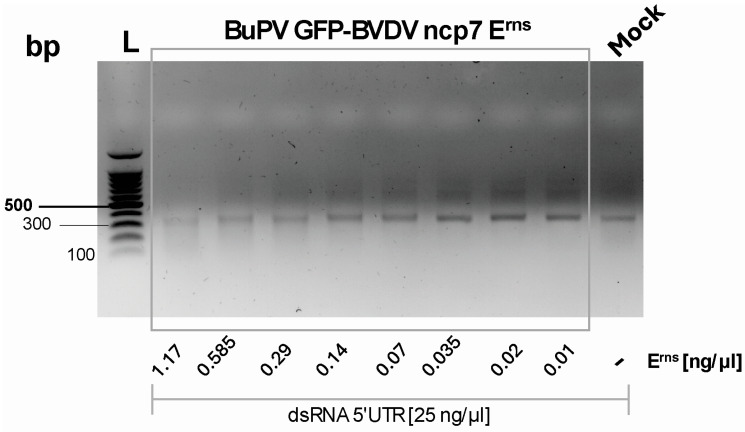
RNase activity assay of GFP-tagged BVDV ncp7 E^rns^. Agarose gel electrophoresis of 25 ng/μL dsRNA following incubation with decreasing concentrations of a 1:2 serial dilution of GFP-BVDV ncp7 E^rns^ in DMEM without FCS. As a negative control, mock represents the diluent without E^rns^.

To assess whether the GFP-tagged BVDV ncp7 E^rns^ retained its RNase activity, we performed a functional assay using supernatants from infected SK-6 cells. The supernatant was processed by sequential ultrafiltration using a 100 kDa molecular weight cut-off (MWCO) membrane to remove infectious particles, followed by a concentration of the E^rns^ protein with a 50 kDa MWCO membrane. The E^rns^ concentration in the resulting retentate was determined by ELISA. Based on these measurements, defined concentrations of the E^rns^-containing preparation were incubated with a 300 bp dsRNA fragment derived from the 5′ untranslated region (5′-UTR) of the BVDV strain Ncp7 for one hour. The reaction mixtures were resolved on a 1% agarose gel to evaluate dsRNA degradation. As shown in Figure 5, increasing concentrations of GFP-E^rns^ led to a progressive reduction in the dsRNA band intensity, demonstrating dose-dependent degradation. These results confirm that the GFP-tagged E^rns^ protein present in the supernatant retains its RNase activity and that fusion to GFP does not impair the enzymatic function of E^rns^.

## 4. Discussion

In this study, we generated and characterized a recombinant Bungowannah virus (BuPV) expressing a GFP-tagged E^rns^ protein derived from BVDV ncp7. This construct was designed to enable real-time visualization and functional analysis of E^rns^ while preserving its biological properties. Our results demonstrate that the fusion of GFP to the N-terminus of E^rns^ is well tolerated, allowing stable expression across multiple viral passages and preservation of enzymatic activity, as confirmed by both structural and functional assays, although replication of the GFP-tagged virus was slower than that of the parental viral stock.

The stability of GFP expression across five sequential viral passages indicates that the recombinant BuPV genome can accommodate and maintain the GFP-E^rns^ fusion without rapid loss or rearrangement of the inserted sequence (Figure 2a). This finding is consistent with the known genetic plasticity of BuPV, which has been exploited in previous work for designing vaccine vectors, including BuPV–BVDV chimeras [20]. Fluorescence microscopy consistently showed strong GFP signals in infected cells from passage 1 through passage 5, and Western blot analysis (Figure 4) confirmed the presence of the intact fusion protein in all passages. The absence of a selective pressure for insert loss under our culture conditions, despite reduced replication efficiency, suggests that the fusion is a neutral or minimally deleterious trait, likely to persist beyond five passages. This genetic stability is a prerequisite for reproducibility in experiments requiring extended virus amplification or repeated infection cycles.

Infection experiments in multiple mammalian cell lines (Figure 2 and Figure 3) confirmed that the recombinant virus retained a broad host range, consistent with prior reports for BuPV [18]. Accordingly, the GFP-tagged virus showed similar infectivity in porcine- (SK6) and bovine-derived cells (MDBK and bovine esophagus) but greater infectivity compared to non-bovine lines (HEK-293a and Vero-E6). This could be related to the exchange of BuPV E^rns^ with BVDV E^rns^, as BVDV is more host-adapted to cattle. The higher infectivity in bovine cells might reflect enhanced interaction of BVDV E^rns^ with bovine heparan sulfate or other bovine host proteins involved in viral assembly for virion-incorporated E^rns^. However, it is in line with the observation [17] that the RNA replication capacity, in addition to the structural envelope glycoproteins, is also of importance for the unique cell tropism of BuPV. Despite measurable attenuation in replication compared to the parental BuPV in all tested cell lines (Figure 2b,c and Figure 3b), the recombinant construct remained functional, detectable, and suitable for studying pestivirus protein trafficking and host–virus interactions. This attenuation may result from the insertion of the relatively large GFP–E^rns^ cassette, which could impair genome packaging efficiency, replication kinetics, or virion assembly. In addition, the heterologous nature of the bovine E^rns^ within a porcine viral backbone may alter protein–protein interactions or host factor compatibility, thereby reducing viral fitness. It is also conceivable that both factors, i.e., the insertion of a bovine E^rns^ and GFP, act synergistically, collectively contributing to the observed attenuation phenotype.

Western blot analysis revealed the presence of two distinct bands reacting with both anti-GFP and anti-E^rns^ antibodies (Figure 4), indicating multiple forms of the GFP-E^rns^ fusion protein. To clarify the identity and composition of these bands, we excised them from the PVDF membrane and subjected them to mass spectrometry (Table 2). The fusion protein was detected in both bands, with a higher number of spectral counts in the lower band. E1, another viral structural protein, was detected only in the lower band, supporting the notion that the two bands reflect different molecular states of the fusion protein. The detection of E1 (4 spectral counts) in the lower band suggests that this band may comprise both the GFP-E^rns^–E1 precursor and a fraction of cleaved GFP-E^rns^ (17 spectral counts) no longer associated with E1. This interpretation is supported by the higher spectral abundance of GFP-E^rns^ relative to E1 in this band. In contrast, the upper band, which shows a further decrease in GFP-E^rns^ abundance and no detectable E1, likely corresponds to the mature, cleaved form of GFP-E^rns^. In addition, the upper band showing altered electrophoretic mobility could reflect a post-translational modification, such as glycosylation, which might reflect the mature version of E^rns^ [33]. Although no glycopeptides were detected in our mass spectrometry dataset, this modification is plausible given that E^rns^ is known to be highly glycosylated and, as such, to be part of the mature virion [34].

A key uncertainty in interpreting our results is that the GFP signal observed in fluorescence microscopy cannot be directly assigned to either the secreted form of E^rns^ or the virion-associated form incorporated during assembly. This distinction is important, as E^rns^ performs different functions depending on its form: the structural protein contributes to virion stability and entry, whereas the secreted protein is implicated in immune modulation. To address this limitation, a recombinant BuPV could be engineered to express fluorophore-tagged E2 protein in addition to GFP-E^rns^ [35]. By labeling E2 with a spectrally distinct fluorophore, it would be possible to track the co-localization of E^rns^ and E2 in real time. Overlapping signals would indicate that E^rns^ is incorporated into assembling virions, whereas the absence of co-localization would suggest that the detected E^rns^ is in its secreted form.

The functionality of the expressed fusion protein was further validated through an RNase activity assay. Supernatants from infected cells were filtrated to remove virus particles and treated to inactivate any residual virus. The concentrated fraction containing free E^rns^ was then incubated with a 300 bp dsRNA fragment derived from the 5′ UTR of BVDV ncp7. Agarose gel analysis showed progressive degradation of the dsRNA with increasing concentrations of GFP-E^rns^ (Figure 5), demonstrating dose-dependent RNase activity. These results indicate that the enzymatic function of E^rns^ is preserved in the fusion construct despite the N-terminal addition of GFP, as was observed with the E^rns^-GFP fusion protein expressed as a single protein [21]. Importantly, this confirms that the GFP tag does not interfere with the catalytic activity of the protein and supports its use in downstream functional assays.

Together, these findings demonstrate that the GFP-tagged BVDV E^rns^ expressed by BuPV retains its essential properties of stability, detectability, and RNase activity. The construct provides a valuable tool for future investigations into E^rns^ biology, including its roles in immune evasion, viral persistence, and secretion dynamics. And with the use of an E^rns^ protein from BVDV, we keep the advantage of retaining the ability to use standard E^rns^ antibodies and ELISAs that are not yet available for BuPV. Beyond this study, additional experiments could explore E^rns^ interactions with host factors or employ advanced tagging strategies, such as a SunTag-based reporter virus, to monitor early infection stages before E^rns^ is secreted or incorporated in a virion [36]. Such approaches could yield new insights into the temporal regulation of E^rns^ trafficking and function. In addition, this virus tagged at the E^rns^ glycoprotein nicely complements other constructs of pestiviruses encoding for fluorescent markers at various other positions, e.g., within the nonstructural proteins [37,38,39], or at the E2 structural protein [35,40] or between the N^pro^ and capsid, which will be cleaved from N^pro^ [41,42], which will facilitate more detailed analyses of the viral replication cycle. Interestingly, Linda virus (LindaV, *Pestivirus* L.), the closest known relative of BuPV, may also represent a promising alternative scaffold for similar genetic manipulations [43]. Given its phylogenetic proximity, it is conceivable that Linda virus shares a comparable degree of genetic plasticity. However, to date, no studies have systematically evaluated the ease of genetic modification of Linda virus, and its suitability as a flexible backbone for reporter virus engineering remains to be determined.

In summary, this study provides the first demonstration that BuPV can stably express a GFP-tagged BVDV E^rns^ over multiple passages while preserving enzymatic function. By combining genetic stability, fluorescent traceability, and preserved functional integrity, this recombinant virus represents a versatile platform for real-time in vitro visualization of E^rns^ dynamics and for advancing our understanding of pestiviral protein biology, with potential applicability in future in vivo studies.

## Figures and Tables

**Table 1:** List of primers used to generate cDNA fragments and TAR vector. Nucleotides in blue and orange represent the overlap with the TAR cloning vector pCC1BAC-His3 and the PacI sequence, respectively. The primers were designed based on the BuPV complete genome sequence from the NCBI Reference Sequence: NC_023176.1.

Primer	Sequence (3′ ⟶ 5′)	Genome Position
BuPV_frag1_F	CAGGGTTTTCCCAGTCACGACTAATACGACTCACTATAGGTATAACGACAGTAGTTCAAGTGTCG	5′UTR-C
BuPV_frag1_R	ACCGGTACCACCAATAGGC	5′UTR-C
BuPV_frag2_F	AGACTCAAGACGGCTTATACCAC	C-E1
BuPV_frag2_R	ACTGATGTCAAAGGTTCCTGGTC	C-E1
Bungo_frag3_F	TCCTTACTGCCCAGTGGCTA	E1-p7
Bungo_frag3_R	ACCACGATCAACAACAGAAGGA	E1-p7
Bungo_frag4_F	AGAACATAGTGGCTCAAGCTGA	p7-NS3
Bungo_frag4_R	TGGTGTGGCACAGTGATATTCA	p7-NS3
Bungo_frag5_F	TCTGAAAGAAGGTGACATGGCA	NS3-NS5A
Bungo_frag5_R	TGGATGGTCAGGTCAGTCGT	NS3-NS5A
Bungo_frag6_F	TGGTTACAGAGAGGCTTACCTA	NS5A-NS5B
Bungo_frag6_R	TTAAAATTTGAGGCCAACAATTTCCA	NS5A-NS5B
Bungo_frag7_F	GTCTACCAAGGAACTGAAAGGTATGT	NS5B-3′UTR
Bungo_frag7_R	CTGCAGGTCGACTCTAGAGGATCTTAATTAAGGGCTTTTTGGAACTGTGCATAG	NS5B-3′UTR

**Table 2:** Summary of mass spectrometry results from PVDF-excised upper and lower bands. Proteins from upper and lower bands detected on the Western blot presented in Figure 4 were excised from the PVDF membrane and analyzed by mass spectrometry. The table summarizes spectral counts, protein identity, background profile, and signal interpretation. GFP-ncp7 E^rns^ was detected in both bands, with higher abundance in the lower band, while E1 was detected only in the lower band. Swine-related proteins dominated the samples as expected. No direct glycosylation evidence was found; however, the results support the hypothesis that the upper band may represent a modified form of the construct.

Feature	Lower Band	Upper Band	Conclusion
GFP-E^rns^ Detection	17 spectral counts	6 spectral counts	Detected in both bands; stronger in lower band
E1 Protein Detection	4 spectral counts	0 spectral counts	E1 present only in lower band
Swine Proteins	Dominant background	Dominant background	Consistent with expression system
Glycosylation	None detected	None detected	Plausible based on gel shift; not confirmed

## Data Availability

The original contributions presented in the study are included in the article; further inquiries can be directed to the corresponding author. The raw data supporting the conclusions of this article will be made available by the authors on request.

## Supplementary Materials

The following supporting information can be downloaded at: https://www.mdpi.com/article/10.3390/v18020263/s1, Table S1: Statistical comparison of viral titers and viral RNA load between BuPV viral stock and GFP-tagged BuPV; Figure S1: Full length uncropped gels for Figure 4.
