# Supplementary material for: GFP-Tagged Erns in Bungowannah Pestivirus: A Tool for Viral Tracking and Functional Studies
Source: Viruses. 2026 Feb 20;18(2):263. doi: 10.3390/v18020263 (PMC12944915; doi:10.3390/v18020263)
Supplement: Supplementary file 1 [file viruses-18-00263-s001.zip › viruses-4129687-supplementary.pdf]

Supplementary Information

GFP-Tagged E^{rns} in Bungowannah Pestivirus: A Tool for Viral Tracking and Functional Studies

Sara Ezzat[1,2,3], and Matthias Schweizer[1,2]

[1] Institute of Virology and Immunology, Laenggass-Str. 122, POB, CH-3001 Bern, Switzerland
[2] Department of Infectious Diseases and Pathobiology, Vetsuisse Faculty, University of Bern, Bern, Switzerland.
[3] Graduate School for Cellular and Biomedical Sciences, University of Bern, Switzerland

		Titer ($TCID_{50}$/ml)		qPCR (C_T value)	
Time point (h.p.i)	Comparison	Significance	Adjusted p-value	Significance	Adjusted p-value
0	BuPV viral stock vs. BuPV GFP-Erns p1	ns	0.852	**	0.0047
	BuPV viral stock vs. BuPV GFP-Erns p2	ns	0.9997	ns	0.0583
	BuPV viral stock vs. BuPV GFP-Erns p3	ns	0.2916	****	<0.0001
	BuPV viral stock vs. BuPV GFP-Erns p4	ns	0.9645	ns	0.8256
	BuPV viral stock vs. BuPV GFP-Erns p5	ns	0.3875	****	<0.0001
6	BuPV viral stock vs. BuPV GFP-Erns p1	*	0.018	ns	0.5542
	BuPV viral stock vs. BuPV GFP-Erns p2	*	0.0291	ns	0.6664
	BuPV viral stock vs. BuPV GFP-Erns p3	*	0.0145	ns	0.0984
	BuPV viral stock vs. BuPV GFP-Erns p4	*	0.0297	ns	0.6521
	BuPV viral stock vs. BuPV GFP-Erns p5	*	0.0175	ns	0.1436
8	BuPV viral stock vs. BuPV GFP-Erns p1	***	0.0008	ns	0.0919
	BuPV viral stock vs. BuPV GFP-Erns p2	*	0.0252	ns	0.6164
	BuPV viral stock vs. BuPV GFP-Erns p3	***	0.0005	*	0.0104
	BuPV viral stock vs. BuPV GFP-Erns p4	**	0.0019	ns	0.0727
	BuPV viral stock vs. BuPV GFP-Erns p5	***	0.0006	ns	0.0697
24	BuPV viral stock vs. BuPV GFP-Erns p1	****	<0.0001	****	<0.0001
	BuPV viral stock vs. BuPV GFP-Erns p2	****	<0.0001	****	<0.0001
	BuPV viral stock vs. BuPV GFP-Erns p3	****	<0.0001	****	<0.0001
	BuPV viral stock vs. BuPV GFP-Erns p4	****	<0.0001	****	<0.0001
	BuPV viral stock vs. BuPV GFP-Erns p5	****	<0.0001	****	<0.0001
48	BuPV viral stock vs. BuPV GFP-Erns p1	***	0.0009	****	<0.0001
	BuPV viral stock vs. BuPV GFP-Erns p2	**	0.0018	***	0.0003
	BuPV viral stock vs. BuPV GFP-Erns p3	**	0.001	****	<0.0001
	BuPV viral stock vs. BuPV GFP-Erns p4	*	0.0102	**	0.0018
	BuPV viral stock vs. BuPV GFP-Erns p5	**	0.0045	**	0.0048
72	BuPV viral stock vs. BuPV GFP-Erns p1	****	<0.0001	****	<0.0001
	BuPV viral stock vs. BuPV GFP-Erns p2	****	<0.0001	***	0.0007
	BuPV viral stock vs. BuPV GFP-Erns p3	****	<0.0001	***	0.001
	BuPV viral stock vs. BuPV GFP-Erns p4	****	<0.0001	ns	0.0719
	BuPV viral stock vs. BuPV GFP-Erns p5	****	<0.0001	*	0.043

Table S1. Statistical comparison of viral titers and viral RNA load between BuPV viral stock and GFP-tagged BuPV (passages 1–5) at different hours post-infection (h.p.i). Columns three and four show comparisons of viral titers ($TCID_{50}$/ml), while the last two columns represent comparisons of viral RNA load (C_T values) determined by qPCR. Viral titers and C_T values were compared using a one-way ANOVA followed by Tukey's multiple-comparison test, performed in GraphPad Prism version 10 for Windows (GraphPad Software, San Diego, CA, USA). Significance is indicated as follows: ns = not significant; *$p < 0.05$; **$p < 0.01$; ***$p < 0.001$; ****$p < 0.0001$. Adjusted p-values are shown for each comparison.

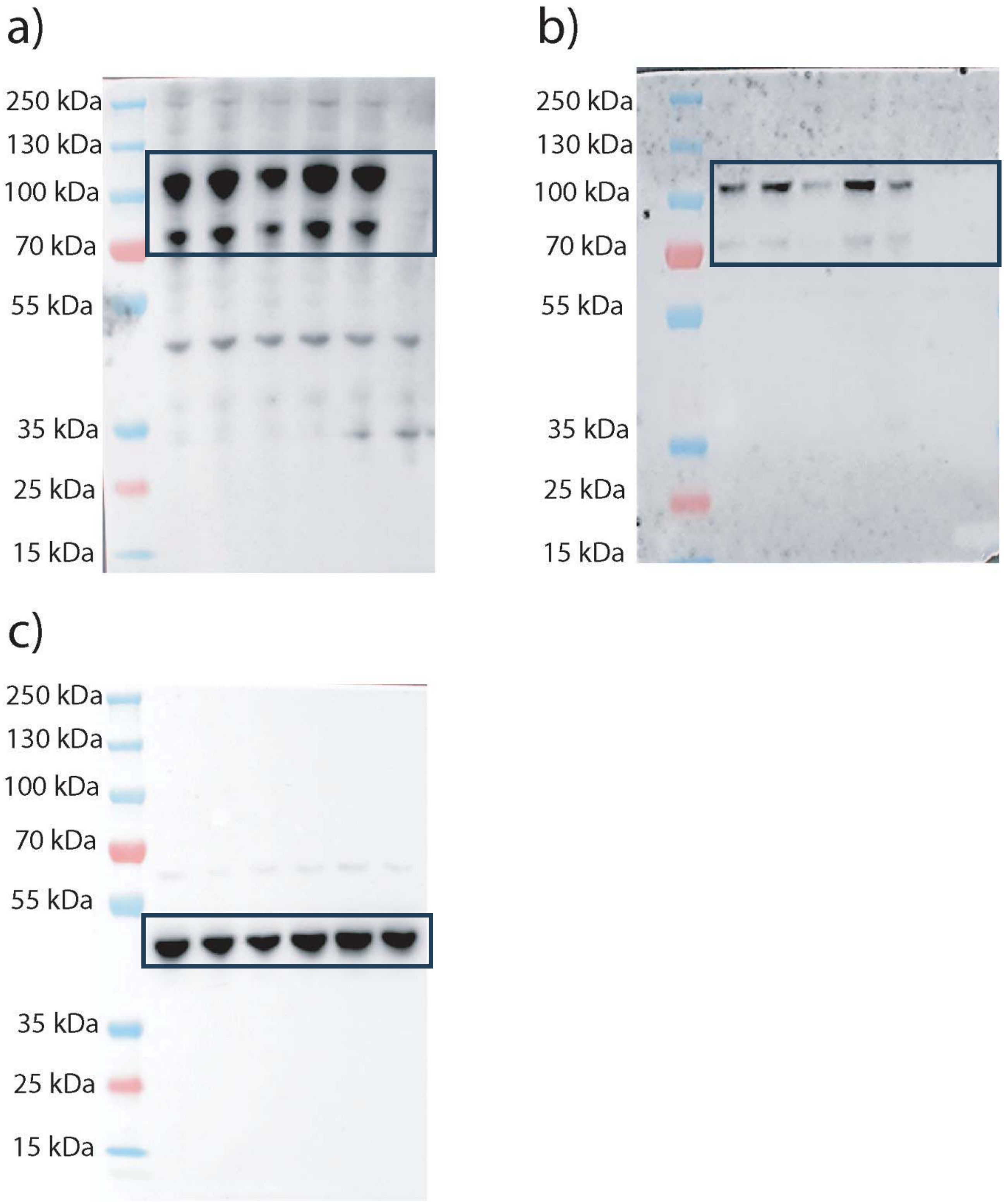

Supplementary Figure S1. Full-length uncropped gels for Figure 4.
Cropped areas used in Figure 4 for the anti-GFP (a), anti-E^{rns} (b) and anti-β actin (c) antibodies are marked with black boxes. The molecular weights for the prestained marker is indicated on the left.
